# Context-dependent behavioural plasticity compromises disruptive selection of sperm traits in squid

**DOI:** 10.1371/journal.pone.0256745

**Published:** 2021-08-30

**Authors:** Noritaka Hirohashi, Noriyosi Sato, Yoko Iwata, Satoshi Tomano, Md. Nur E. Alam, Lígia Haselmann Apostólico, José Eduardo Amoroso Rodriguez Marian

**Affiliations:** 1 Department of Life Sciences, Shimane University, Shimane, Japan; 2 School of Marine Science and Technology, Tokai University, Shizuoka, Japan; 3 Atmosphere and Ocean Research Institute, University of Tokyo, Chiba, Japan; 4 Departamento de Zoologia, Instituto de Biociências, Universidade de São Paulo, São Paulo, Brazil; University Hospital of Münster, GERMANY

## Abstract

Sperm morphology is generally uniform within a species due to selective pressures that act to achieve better fertilization outcomes under postcopulatory competitive circumstances. Therefore, polyandry that intensifies post-mating sperm competition should constrain intraspecific sperm polymorphism. Contrary to this paradigm, we previously found that a polyandrous squid, *Heterololigo bleekeri*, produces dimorphic eusperm (flagellum length dimorphism; FLD), which is closely associated with alternative reproductive tactics (ARTs); large males (consorts) transfer their spermatophores inside the female’s mantle cavity, while small males (sneakers) do so outside the mantle. Thus, FLD was considered as the consequence of different insemination strategies that arise from different modes of sperm competition, sperm storage and the fertilization environment. However, in other squid species showing ARTs, the choice of mating behaviour is rather conditional (i.e., switching mating tactic between consorts and sneakers), which poses the question of whether sperm FLD could have evolved. Here, we investigated five species in the family Loliginidae that exhibit ARTs and found that all species showed sneaker-biased FLD. However, in a species with conditional ARTs, we found FLD rather ambiguous and the testicular somatic index to be nearly continuous among individuals at transitional state, suggesting that plasticity in mating behaviour compromises the disruptive selection on a sperm morphological trait.

## Introduction

In circumstances where intense male–male competition occurs, male individuals are not given equal mating opportunities. Consequently, small males often exhibit alternative mating tactics, such as sneaking or female mimicking, to approach females and compensate for their lower chances of winning physical contests against larger competitors, thereby maximizing their reproductive success [1, 2]. In accordance with these behavioural tactics, small males also invest more reproductive resources on ejaculate traits than larger males, primarily in quantity (relative or absolute sperm number) but also occasionally in quality (e.g., sperm swimming performance) [3]. Because the individual benefits of these behavioural and ejaculate traits on reproductive success depend on social status/agonistic ranks that can change during growth or aging [4], sperm traits should ideally be developed or switched into fitness optima according to their prospects. However, in reality, males’ reproductive success largely relies on their current social contexts arising instantaneously from intra- and intersexual interactions, therefore behavioural plasticity, but not extremely-developed ejaculate traits, would be favoured due to a time constraint for spermatogenesis. Nevertheless, the extent to which such plasticity influences developmentally regulated alternative sperm traits remains unexplored.

Squids of the family Loliginidae are excellent models to investigate this question because they show sophisticated alternative reproductive tactics (ARTs) by which small males, known as “sneakers”, produce longer spermatozoa [5, 6] and attempt sneaky (extra-pair) copulation to deposit sperm packages (spermatophores) into female arm bases where the seminal receptacle is located [7]. In contrast, large “consort” males have shorter spermatozoa and copulate via “male-parallel” pair-bonding to deposit their spermatophores near the internal female oviduct, resulting in two different locations and modes of sperm storage within a female. In addition, plasticity in mating behaviour by male individuals is common in some species [8–12] while it is either rare or absent in others [5, 13]. For example, intermediate-sized males of *Doryteuthis pleii* in Brazilian waters make a tactical decision at each mating opportunity based on the female reproductive context; pair bonding at egg laying and sneaking when further from spawning [12]. In Caribbean reef squid *Sepioteuthis sepioidea*, sneaking is a common practice for early adult males but changes to pair bonding (consortship) as they grow [10]. In *S*. *lessoniana*, male individuals display either pair bonding (“male-parallel”) or sneaking (“male-upturn”) according to relative female size [9], which is determined primarily by the female’s choice to consent to or reject one male-mating posture over another [11]. In contrast, although behavioral flexibility was observed in captivity in Japanese spear squid *Heterololigo bleekeri* [14], available evidence from field observations [13] and anatomical investigations of attached spermatangia on female [5] does not support the assumption that individuals in the wild populations also exhibit phenotypic plasticity in male mating behaviour. In addition, from an anatomical point of view, male consorts do not become sexually mature until they reach a certain growth point in body size, and at this point, they are much bigger than typical male sneakers. This phenomenon is also observed in *Loligo forbesi* [15]. Hence, squid ARTs display a broad spectrum of adaptive traits including a variable intensity of phenotypic plasticity with a complex repertoire of behaviour, morphology and physiology [16]. In light of these interspecific variables, here we address the evolutionary consequences of sperm flagellum length dimorphism (FLD) in closely related species with different levels of tactical plasticity.

## Materials and methods

### Sample collection and male phenotype determination

Animals and their collected locations were as follows: *Sepioteuthis lessoniana* (the Amami Islands, Kagoshima Pref., off Sakai port, Tottori Pref. Japan), *Heterololigo bleekeri* (Tsugaru Strait, Hokkaido, Japan), *Uroteuthis edulis* (the Oki Islands and Tsushima Strait, Nagasaki, Japan), *Doryteuthis pleii* (São Sebastião Island, São Paulo, Brazil), and *Loligo reynaudii* (Port Elizabeth, South Africa). The collection methods were either by commercial set-net and trawling fisheries, or luring and net trapping by researchers. Animals obtained from commercial fishing were already dead; therefore, dissections and measurements were conducted immediately. Male type determination was carried out by observing the spermatangium morphology; the tube-like or teardrop-like structure as consorts or sneakers, respectively [17]. Some raw data of *D*. *pleii* and *L*. *reynaudii* obtained from previous studies [18, 19] were reexamined with different parameters. All procedures performed in the studies were in accordance with the ethical standards of the Animal Research Committee of Shimane University (ARCSU) and animal experiments were approved by ARCSU (MA2-2).

### Sperm size and other measurements

Measurement of sperm flagellum length (FL) was carried out as described previously [5]. Briefly, sperm were released from the spermatophores collected from the male’s spermatophoric sac or from the spermatangia attached to the female body. Sperm were fixed with 4% formaldehyde (nacalai tesque, INC., Kyoto, Japan) in seawater and stored at room temperature. Aliquots of formaldehyde-fixed sperm were mounted on a slide glass and viewed under a microscope (Nikon TE-2000) with a ×20 objective lens. Images were captured with a USB camera and FL was measured with ImageJ software (National Institutes of Health). Sample sizes for each species were as follows: *L*. *reynaudii*: 22 consorts, 14 sneakers; *H*. *bleekeri*: 30 consorts, 30 sneakers; *U*. *edulis*: 52 consorts, 33 sneakers*; D*. *pleii*: 30 consorts, 30 sneakers and *S*. *lessoniana*: 8 consorts, 8 sneakers. Twenty spermatozoa per male were measured and data were deposited in S1 File.

## Results

**Fig 1:**
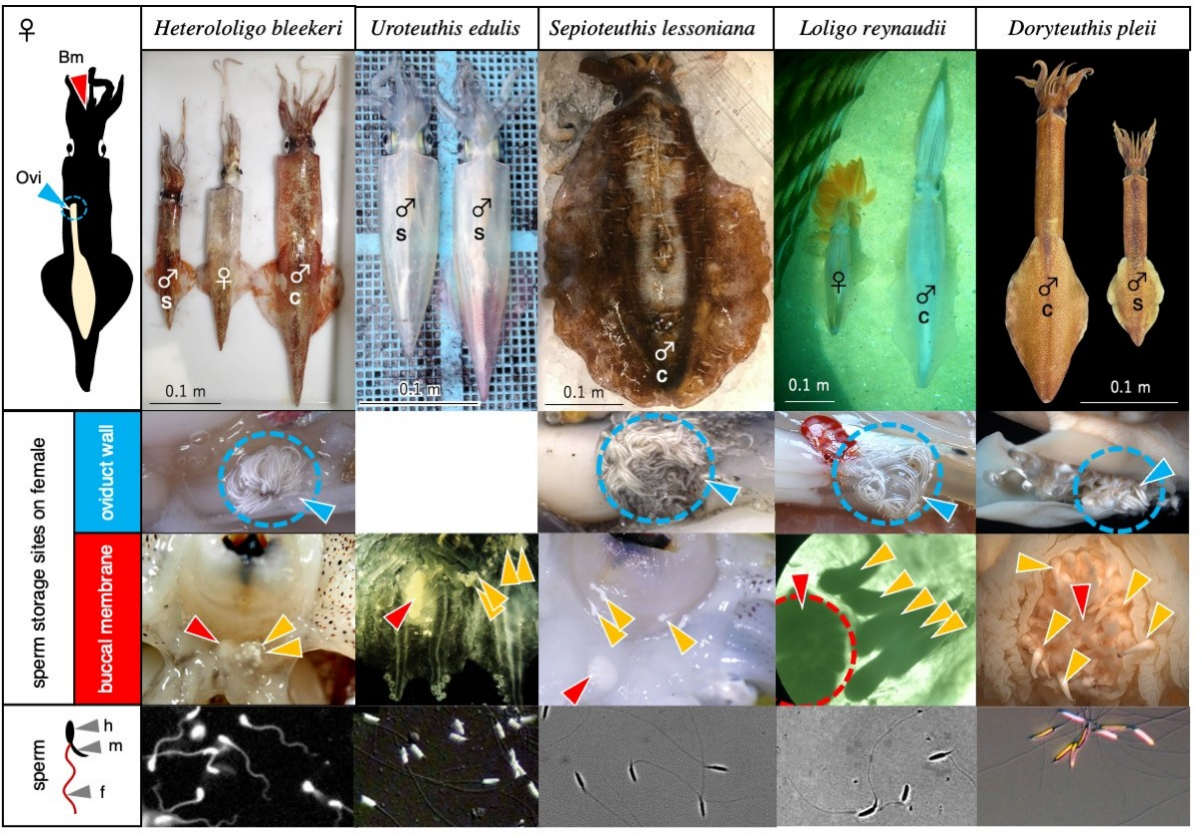
Comparative anatomy of female’s sperm storage sites in squids. *Top* row, a diagram of female’s sperm storage sites, i.e., on the oviduct wall (Ovi) and buccal membrane (Bm), and five species in the family Loliginidae used in this study. *Second* and *third* rows, the spermatangia attached to the oviduct wall (*blue* arrowheads) and buccal membrane (*yellow* arrowheads). The seminal receptacle (*red* arrowhead) is located on the buccal membrane. *Bottom* row, representative images of spermatozoa retrieved from the spermatangia. Photographs from the oviduct wall and buccal membrane of *D*. *pleii* were originally published in [30] and are reproduced here with permission. s, sneaker; c, consort; h, head; m, midpiece enriched with mitochondria; f, flagellum.

**Fig 2:**
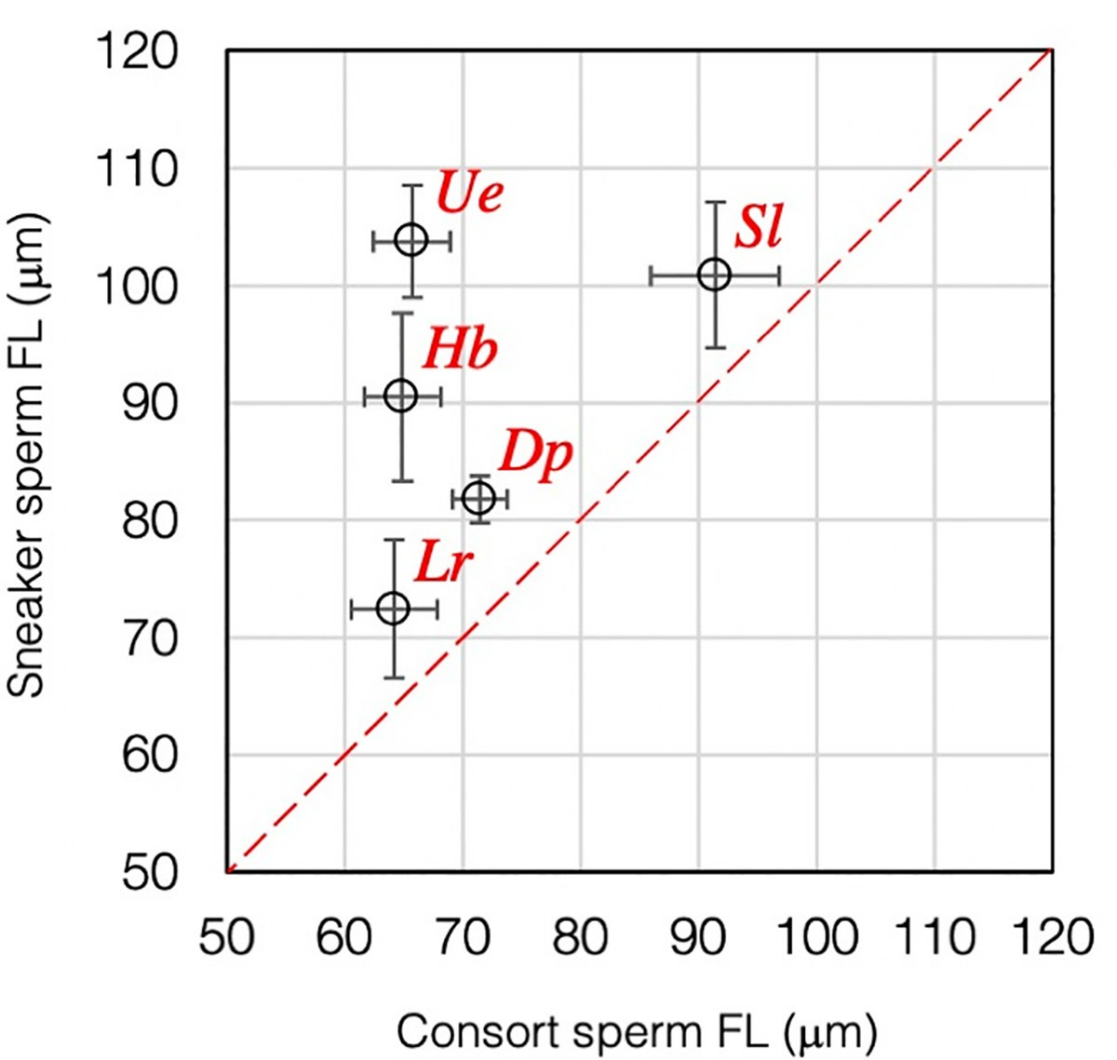
Comparison of sperm flagellum length between consorts and sneakers in five coleoid squids exhibiting Alternative Reproductive Tactics (ARTs). Intraspecific sperm dimorphism between sneakers and consorts in five species of loliginid squids. Each data point represents the mean ± SD with 8–52 male individuals (see *Materials and Methods*). Hb, *H*. *bleekeri*; Dp, *D*. *pleii*; Ue, *U*. *edulis*; Sl, *S*. *lessoniana*; and Lr, *L*. *reynaudii*.

**Fig 3:**
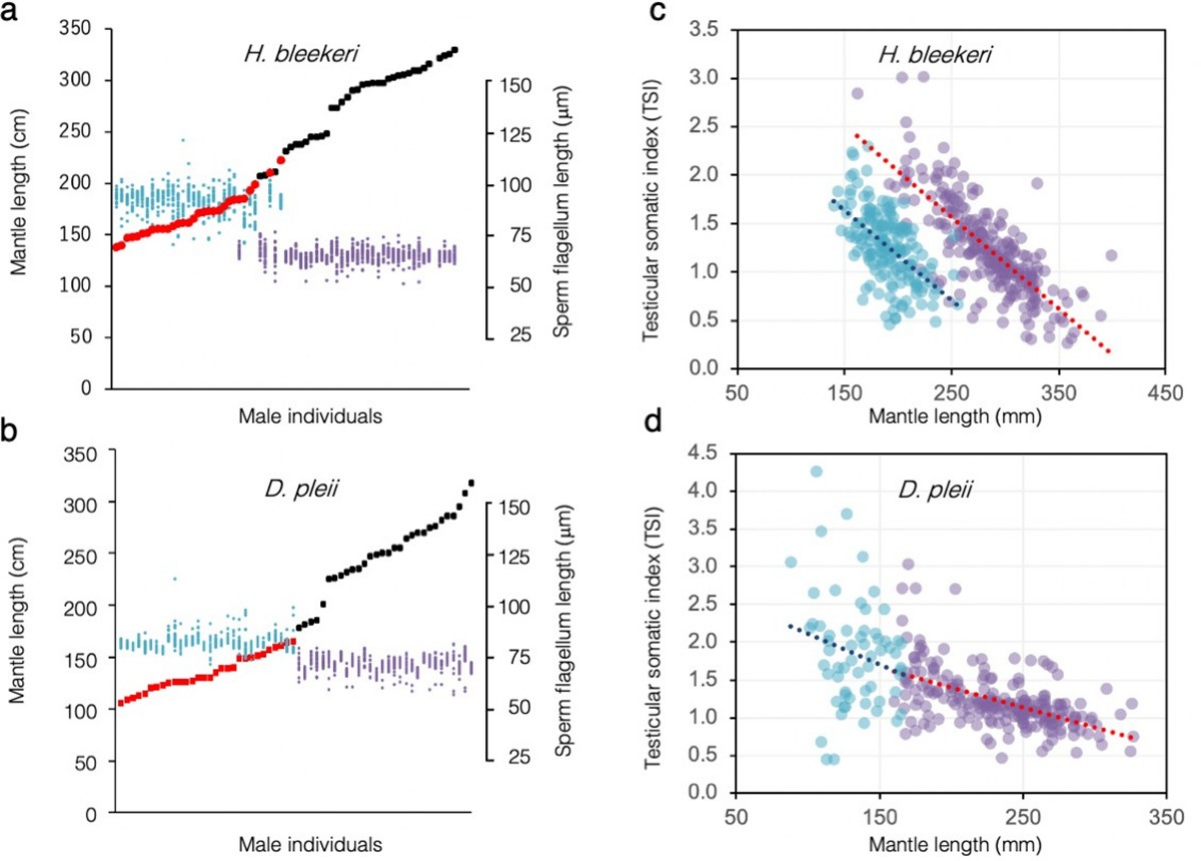
Changes in sperm FL and reproductive indices among individuals associated with size distribution in *H*. *bleekeri* and *D*. *pleii*. Male individual measurements in FL are arranged in order of increasing mantle length. For each male individual in *H*. *bleekeri* (a) and *D*. *pleii* (b), ML (*closed circles*) and FL values of 20 spermatozoa (*scattered dot*s) have been plotted. The colour code represents sneaker males (*cyan* and *red*) or consort males (*purple* and *black*). Relationship between the mantle length and relative testis mass (testicular somatic index; TSI) in sneaker (*cyan*) and consort (*purple*) individuals are plotted for *H*. *bleekeri* (c) and *D*. *pleii* (d).

We performed an intraspecific comparison of sperm FL between sneaker and consort males in five species displaying ARTs (Fig 1). In all cases, the FL was longer in sneakers than in consorts (Fig 2). However, intraspecific, inter-tactic differences in the FL varied significantly between species; for example, the difference was substantial in *U*. *edulis* (1.6-fold) and *H*. *bleekeri* (1.4-fold), whereas it was small in *D*. *pleii* (1.1-fold) and *S*. *lessoniana* (1.1-fold). We then compared two Loliginidae species, *H*. *bleekeri* and *D*. *pleii*, exhibiting contrasting levels in FLD. When within-individual variables of sperm FL were plotted across different size classes of mature males arranged in order of increasing mantle length, we found that FLD was much clearer in *H*. *bleekeri* than in *D*. *pleii* (Fig 3A and 3B). Furthermore, when intraspecific, intra-tactic analysis was carried out in both species, we found a negative linear corelation between male mantle length (ML) and TSI (Fig 3C and 3D). However, in *H*. *bleekeri*, these correlations were found to be discontinuous between sneakers and consorts (Fig 3C), whereas, in *D*. *pleii*, such the ML-based discontinuity in TSI was invisible (Fig 3D).

## Discussion

Although morphological diversification of sperm across animal taxa is known, intraspecific variation of sperm morphology is limited because strong selection should favour the fittest sperm in a given set of physiological and competitive conditions [20]. It has also been shown in some animal taxa that interspecific variability in sperm FL is positively correlated with sperm competitiveness, which is determined mostly by relative testis size, a widely used benchmark for male promiscuity (internal fertilizers) or gonad expenditure (broadcast spawners), and therefore the level of sperm competition [21–23]. Parker [24] recognized for the first time that sneakers are subject to higher sperm competition risks than guarders (consorts) and proposed that greater reproductive expenditures should be manifested in sneakers than in guarders (the sneak-guard model). Consequently, empirical studies addressing this hypothesis have been carried out with a great variety of animal groups including a large number of bony fishes [25–27], insects [28] and mammals [29]. Surprisingly, although the sneak-guard model is largely unambiguously supported, sneaker-favoured reproductive expenditures on ejaculate traits, if any, are observed mostly in sperm number and rarely in sperm size or performance (longevity and swimming speed) [3].

However, our current and previous [5, 6] results of squid species with ARTs, unlike most other animals, demonstrated clear sneaker-biased FLD (Fig 2), which cautions us against dealing the squid sneaker-consort system as a representative of general ARTs models. First, squid utilize two separate insemination sites—internal and external to the female body cavity—generating completely different sets of adaptive optima in sperm storage condition and physico-chemical fertilization environment. Second, the squid sneaker tactic is unlikely to be strictly an “alternative” method against a consort tactic from behavioural and ontogenetic aspects. A number of squid and cuttlefish species employ only sneaker-like copulations (i.e., absence of pair-bonding or mate guarding) and the female seminal receptacles serve exclusively/primarily to store these sperm [7]. Third, in some species with ARTs, males choose one or the other tactic at mating, depending on the conditions of potential mates but not of rival males [9, 11], and males of *S*. *sepioidea* always attempt sneaky copulations when they are young [10], suggesting independence of a consort tactic. Given that squid ARTs involve multimodality in male dimorphism, the ART-associated sperm FLD could have arisen from one or more such different modes, making it difficult to identify a key evolutionary force (or forces) critical for driving disruptive selection on sperm traits.

Nevertheless, the trend of close association between male insemination tactic and sperm FLD is evident in all examined Loliginidae species with ARTs (Fig 2). Because these intraspecific, intertactic differences would have arisen from the disruptive selection by which antagonistic ejaculate traits can evolve, species variation in sperm FLD might have arisen from interspecific differences in intensity of tactical conflict. We speculate that intensity of tactical conflict can be influenced by the degree of behavioural flexibility; that is, if male mating behaviours are conditional or transformable, diverging ejaculate traits that maximize male reproductive fitness for each tactic would tend to be less developed. Based on this assumption, we compared two closely related species, *H*. *bleekeri* and *D*. *pleii*, exhibiting contrasting levels in FLD. The results showed clear ML-based discontinuities in FL and TSI in *H*. *bleekeri* (Fig 3A and 3C). This suggests that the binary “sneaker-or-consort” fate decision might occur before males become sexually mature, which is consistent with previous findings that *H*. *bleekeri* males exhibit a positive linear relationship in size between spermatophore length and ML, a correlation that is discontinuous between sneakers and consorts [13]. However, in *D*. *pleii*, such ML-based discontinuities in sperm FL and TSI are absent (Fig 3B and 3D), which is consistent with previous observations that adult males with intermediate body size (132–178 mm ML) could change their mating tactics flexibly in response to female spawning behaviours [12]. Thus, an ontogenetic transition from sneaker to consort might occur within the same individuals [10, 12, 16]. Such flexibility in male mating behaviour was also observed in *S*. *lessoniana* under experimental conditions where behavioural choice in ARTs was primarily based on the relative body size of the mating partner [9, 11]. These results, combined with our previous findings [12, 13] and others [9, 11], lead us to speculate that context-dependent behavioural plasticity at mating attenuates disruptive selection on tactic-specific alternative morphs and hence allows the emergence of intermediate phenotypes that could have maladaptive—or moderate—fitness for each mating tactic.

Most squid species grow quickly and die within 2 years [7]. Consequently, so-called semelparous reproduction can occur only once and briefly under a high-competition regime. Nevertheless, extremely high degrees of variation in growth rates and body sizes within a population allow individuals to select different strategies for maximizing their mating opportunities, possibly resulting in two different evolutionary trajectories: early ontogenetic decision (*H*. *bleekeri*) and phenotypic plasticity (*D*. *pleii*). Interestingly, this difference could be extended to endocrine control, that is, *H*. *bleekeri* males may cease their growth after becoming a sneaker, while *D*. *pleii* may continue to grow even after reaching sexual maturity. Despite the close similarities in life histories and reproductive strategies between these two species, what has driven their diverging evolutionary pathways remains to be determined.

## Supporting information

S1 File (DOCX)
