# Supplementary material for: Context-dependent behavioural plasticity compromises disruptive selection of sperm traits in squid
Source: PLoS One. 2021 Aug 30;16(8):e0256745. doi: 10.1371/journal.pone.0256745 (PMC8404977; doi:10.1371/journal.pone.0256745)

S1 File

# Context-dependent behavioural plasticity compromises disruptive selection of sperm traits in squid

Noritaka Hirohashi^1*^, Noriyosi Sato^2^, Yoko Iwata^3^, Satoshi Tomano^3^, Md Nur E Alam^1^, Lígia Haselmann Apostólico^4^, José Eduardo Amoroso Rodriguez Marian^4^

**1** Department of Life Sciences, Shimane University, Shimane, Japan.

**2** School of Marine Science and Technology, Tokai University, Shizuoka, Japan

**3** Atmosphere and Ocean Research Institute, University of Tokyo, Chiba, Japan

**4** Departamento de Zoologia, Instituto de Biociências, Universidade de São Paulo, São Paulo, Brazil

^*^hiro@life.shimane-u.ac.jp

^*^Corresponding to NH.

**Key words:** sperm competition, alternative reproductive tactics, cephalopods, sneakers, behavioral plasticity, disruptive selection, sperm flagellum length dimorphism


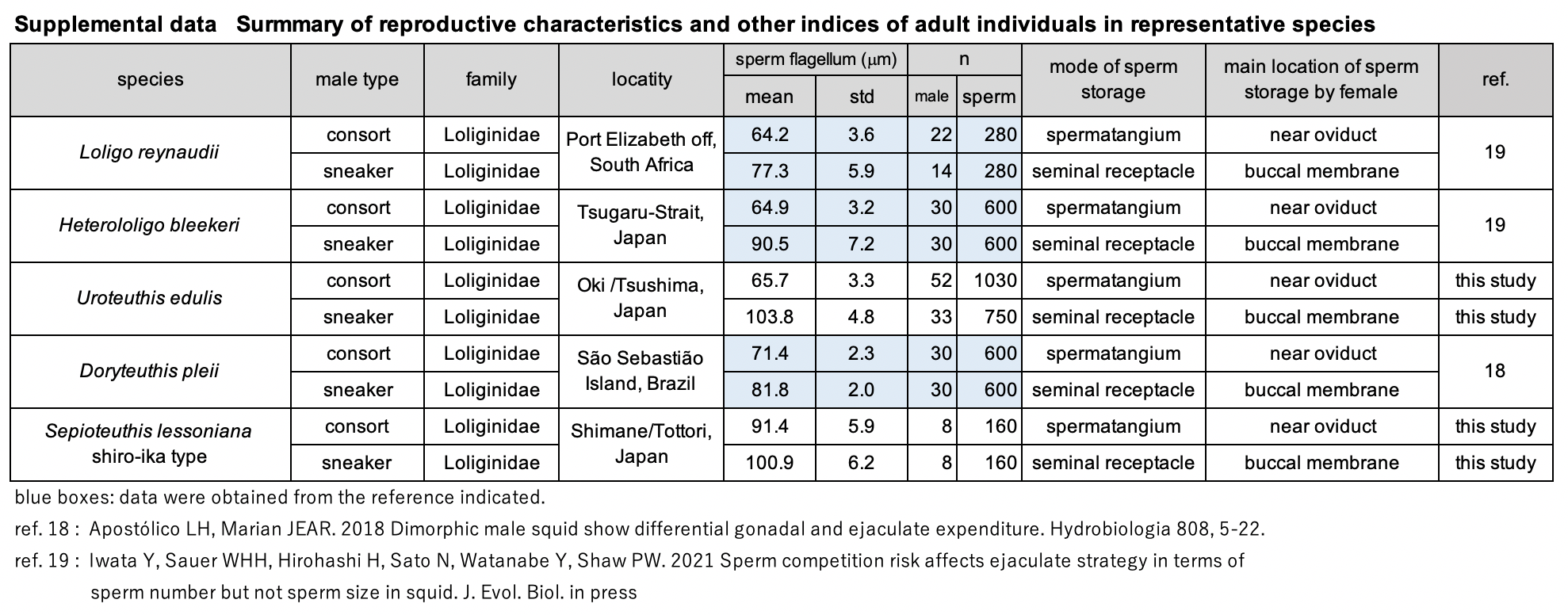

Supplement: S1 File — (DOCX) [file pone.0256745.s001.docx]
